# Southern African HIV Clinicians Society guideline on the management of non-tuberculous mycobacteria in people with HIV

**DOI:** 10.4102/sajhivmed.v25i1.1657

**Published:** 2024-10-23

**Authors:** Halima Dawood, Lauren Richards, Keeren Lutchminarain, Arifa Parker, Camilla Wattrus, Nosisa Sipambo, Jeremy Nel, Thandekile Manzini, Kogieleum Naidoo

**Affiliations:** 1 Infectious Diseases Unit, Department of Internal Medicine, Grey’s Hospital, Pietermaritzburg, South Africa; 2 Centre for the AIDS Programme of Research in South Africa (CAPRISA), Nelson R Mandela School of Medicine, University of KwaZulu-Natal, Durban, South Africa; 3 Division of Infectious Diseases, Department of Internal Medicine, Faculty of Health Sciences, University of the Witwatersrand, Johannesburg, South Africa; 4 National Institute of Communicable Diseases (NICD), Division of the National Health Laboratory Service (NHLS), Johannesburg, South Africa; 5 Unit for Infection Prevention and Control, Department of Medicine and Health Sciences, Stellenbosch University, Cape Town, South Africa; 6 Division of Infectious Diseases, Department of Medicine and Health Sciences, Stellenbosch University, Cape Town, South Africa; 7 Southern African HIV Clinicians Society, Cape Town, South Africa; 8 Infectious Diseases Unit, Department of Paediatrics and Child Health, Faculty of Health Sciences, University of the Witwatersrand, Johannesburg, South Africa; 9 Infectious Diseases Unit, Department of Internal Medicine, Faculty of Health Sciences, Dr George Mukari Academic Hospital, Sefako Makgatho Health Science University, Tshwane, South Africa; 10 Centre for the AIDS Programme of Research in South Africa (CAPRISA), Medical Research Council (MRC), Durban, South Africa; 11 Nelson R Mandela School of Medicine, University of KwaZulu-Natal, Durban, South Africa

## Introduction

Non-tuberculous mycobacteria (NTM), previously also known as mycobacteria other than tuberculosis (MOTT), comprise all mycobacterial species other than those that cause tuberculosis (TB). They are a diverse group of bacteria that are acquired mainly from contaminated environmental sources through aerosols or ingestion, and generally cause infections whose presentations may overlap with *Mycobacterium tuberculosis* complex (Mtb). Disease caused by NTM is seen particularly in people with either structural lung disease or impaired immunity, including HIV, but can sometimes occur in individuals with no obvious risk factors.^1^ Given the high burden of HIV and TB in Southern Africa (SA) and the diversity of species, it is important to be able to clinically evaluate, diagnose and manage NTM in people with HIV (PWH).

This guideline aims to support clinicians to recognise, diagnose and treat NTM in PWH in the SA context, taking differences in resource availability into account. Expert advice should be sought for complex cases. Recommendations may evolve as new information emerges and it is important to consult the most updated guidance.

## Epidemiology

The incidence and prevalence of NTM cases, including strain distribution, is highly variable geographically.^2,3,4^ *Mycobacterium avium* complex (MAC) is an NTM species complex, requiring genotypic testing for species-level differentiation. MAC includes *Mycobacterium avium* and *Mycobacterium intracellulare,* with *Mycobacterium chimaera* having been recently added to the complex.^5^ MAC are the most frequently isolated species in respiratory samples,^6^ and the majority of NTM disease is caused by MAC in the immunosuppressed.^7^ *Mycobacterium abscessus* complex is typically associated with significant immunocompromise.^6,8^ Other common respiratory NTM species include *Mycobacterium kansasii, Mycobacterium malmoense* and *Mycobacterium xenopi*.

Globally, the prevalence of NTM is increasing, including in the United States,^9,10^ Australia,^11^ and Canada.^12^ However, there are few studies documenting the occurrence of NTM in SA, including in PWH. In a Free State study that excluded PWH, NTM isolates were found in the sputum of 27% of a gold-mining workforce, two-thirds of whom had new pulmonary cavitation.^13^ *M. kansasii* had the highest prevalence, followed by *Mycobacterium scrofulaceum, M. avium* and *M. abscessus*. An observational study that included PWH,^14^ also in a gold-mining workforce, found that MAC, *Mycobacterium gordonae* and *M. kansasii* were the most common species found in sputum samples. Those with MAC tended to be more symptomatic and had higher rates of HIV. In a study in KwaZulu-Natal, 200 respiratory specimens obtained from patients with suspected NTM infection were submitted to a central TB laboratory. Of these, 133 (65%) cultured an NTM, with MAC being the most common isolate, accounting for 76 (57.2%) cases.^15^

A systematic review and meta-analysis of NTM pulmonary samples in the sub-Saharan region^16^ found a colonisation prevalence of 7.5%, with MAC being the most frequent coloniser. Of those with pulmonary disease however, *M. kansasii* predominated, constituting nearly 70% of cases. The authors hypothesised that this unusual finding might be attributable to overrepresentation of samples from miners in SA studies. Globally, and in the rest of sub-Saharan Africa, MAC is the major cause of pulmonary NTM disease. In the study, there were high rates of concomitant HIV (40.5%) and a previous history of pulmonary TB (32.4%).

In disseminated disease, MAC is again the most common NTM isolated, followed more distantly by *M. kansasii*, and the rapidly growing mycobacteria.^17^ There is likely considerable geographical heterogeneity in the prevalence within PWH in SA, with a point prevalence of up to 10% having been described in patients with CD4 counts < 100 cells/µL.^18^

In children with HIV (CWH), NTM disease is mostly disseminated. It tends to occur in children older than 5 years, with severe immunosuppression (CD4 < 100 cells/µL).^19,20^ In the United States, antiretroviral therapy (ART) has significantly reduced the incidence of disseminated NTM, from 1.3 cases per 100 person years pre-2004 to 0.2 cases per 100 person years after 2004.^21^ NTM disease in CWH has a high mortality, with a rate of 60% in one case series.^19^ This was likely as a result of delays in diagnosis and institution of definitive treatment, in addition to the disease itself.

There is otherwise little literature on NTM specifically in PWH in sub-Saharan Africa.

## Clinical presentation of non-tuberculous mycobacteria in people with HIV

Although all forms of mycobacterial disease are seen in PWH, the incidence of NTM infections is not directly increased in PWH, apart from disseminated NTM disease and focal lymphadenitis immune reconstitution inflammatory syndrome (IRIS).^7^ NTM infections are variable in their manifestations but usually cause one of five syndromes:

- **Disseminated disease:** Occurs almost exclusively in the severely immunocompromised, and especially in patients with advanced HIV.
- **Pulmonary disease:** Usually occurs in patients with an underlying lung disease such as chronic obstructive pulmonary disease, silicosis, bronchiectasis or previous TB. MAC and *M. kansasii* are typical pathogens, and pulmonary disease has to be differentiated from both Mtb and colonisation.^22^ Isolated pulmonary disease is rare in CWH.^23^
- **Local lymphadenitis:** Occurs in immunocompetent patients. This is often in the cervical region, is usually seen in children, and is typically caused by MAC or *M. scrofulaceum*. In PWH, it usually reflects an IRIS reaction and may involve any lymph node region.
- **Skin and soft tissue infections:** Usually from direct inoculation (which may include inadvertent nosocomial or surgical inoculations). Typical pathogens are *M. marinum* (‘fish-tank granuloma’ with water exposure), *M. ulcerans* (‘Buruli ulcer’ in tropical regions), and the rapidly growing mycobacteria, *Mycobacterium fortuitum, M. abscessus*, and *M. chelonae*.
- **Central catheter-related infections:** Typically caused by one of the rapidly growing mycobacteria.

Table 1 summarises the most common NTM species found in PWH for the various clinical presentations of the disease.^6,8,24^

**TABLE 1:** Common non-tuberculous mycobacteria species found in people with HIV.

Clinical presentation	Species
Disseminated disease	MAC† (usually *Mycobacterium avium*) *Mycobacterium kansasii*
Pulmonary disease	MAC† *Mycobacterium kansasii* *Mycobacterium abscessus*
Local lymphadenitis	MAC† *Mycobacterium scrofulaceum* *Mycobacterium malmoense* *Mycobacterium lentiflavum* *Mycobacterium bohemicum*
Skin and soft tissue infections	Rapidly growing NTM‡ *Mycobacterium marinum*

*Source*: Gopalaswamy R, Shanmugam S, Mondal R, Subbian S. Of tuberculosis and non-tuberculous mycobacterial infections – A comparative analysis of epidemiology, diagnosis and treatment. J Biomed Sci. 2020;27:1–17. https://doi.org/10.1186/s12929-020-00667-6; Hoefsloot W, Van Ingen J, Andrejak C, et al. The geographic diversity of nontuberculous mycobacteria isolated from pulmonary samples: An NTM-NET collaborative study. Eur Respir J. 2013;42(6):1604–1613. https://doi.org/10.1183/09031936.00149212; and Martiniano SL, Nick JA, Daley CL. Nontuberculous mycobacterial infections in cystic fibrosis. Clin Chest Med. 2022;43(4):697–716. https://doi.org/10.1016/j.ccm.2022.06.010

MAC, *Mycobacterium avium* complex; NTM, non-tuberculous mycobacteria.

† , MAC includes *Mycobacterium avium, Mycobacterium intracellulare, Mycobacterium chimaera*;

‡ , Rapidly growing NTM includes *Mycobacterium fortuitum, Mycobacterium abscessus, Mycobacterium chelonae*.

### Disseminated disease

In the SA context, disseminated disease is overwhelmingly associated with HIV. The risk is correlated with CD4 count, with HIV-associated disseminated disease most often occurring at CD4 counts < 50 cells/µL, and only seldom at CD4 cell counts of > 100 cells/µL.^7,25^

Disseminated NTM infection is predominantly caused by MAC although it does not appear that there are any important differences in presentation between the species. Species other than MAC are too sparsely represented in most case series to draw firm conclusions.

Disseminated disease usually presents in a non-specific manner, with non-specific constitutional symptoms such as persistent or recurrent fever, night sweats, weight loss, and anorexia being prominent. CWH may also present with failure to gain weight, persistent diarrhoea, and persistent or recurrent abdominal pain.^19,20^ Where there are localising signs, these are often pulmonary (cough) or gastrointestinal (chronic diarrhoea), likely reflecting the two portals of entry of the organisms prior to dissemination.^25,26^ Respiratory symptoms are uncommon in CWH who have disseminated disease.^23^

Hepatomegaly, splenomegaly, and/or lymphadenopathy are also frequently seen, perhaps reflecting the organism’s predilection to proliferate within macrophages and histiocytes. When present, lymphadenopathy is seldom as prominent as seen with the localised lymphadenopathy forms of the disease. Oddly, involvement of the central nervous system in disseminated disease is very rare, and much less common than is seen with Mtb.^27^

Laboratory findings are non-specific. In adults, anaemia is almost always present, and an elevated alkaline phosphatase (ALP) is seen in approximately a third of patients with disseminated disease.^28,29^ An excessively high ALP (20–40 times the upper limit of normal) without elevation of other hepatic enzymes occurs in 5% of patients with MAC. The pathogenesis of this is unclear but may involve interference with enzyme metabolism.^28^

In CWH, laboratory abnormalities include anaemia, leukopaenia, and thrombocytopenia.^19,20^ Although children with disseminated NTM usually have normal serum chemistries, some children may have elevated ALP or lactate dehydrogenase levels. Other diagnoses such as Mtb, disseminated fungal disease, nocardiosis, and malignancies must be considered because of the non-specific nature of the symptoms, signs and laboratory findings.

Biopsy specimens typically reveal histocytes packed with acid-fast organisms (on average far more numerous than typically seen with TB), surrounded by little to no granuloma formation.^26,28^

IRIS reactions are well described following disseminated disease (see section immune reconstitution inflammatory syndrome). Chylous ascites can occur either as part of disseminated disease, or as an IRIS reaction.^30^ The presumed pathogenesis is obstruction of the lymphatic system by either enlarged lymph nodes or lymph node fibrosis.^31^

### Lymphadenitis

When local NTM lymphadenitis occurs in the context of PWH (as opposed to lymphadenitis as part of disseminated disease), it usually reflects an IRIS reaction. Both unmasking and paradoxical IRIS reactions can occur. Any lymph node group can be involved, including abdominal, mediastinal or cervical nodes.^32,33^ Leucocytosis appears common, in addition to systemic features.^33^

## Diagnosis

### Diagnostic criteria for pulmonary non-tuberculous mycobacteria in adults

For a reliable and accurate diagnosis of NTM pulmonary disease, the diagnostic criteria include a combination of clinical, radiographic, and microbiologic criteria.^2,34^ It is important to note that NTM can be isolated from respiratory specimens because of environmental contamination or due to respiratory colonisation with NTM in the absence of disease.^34^ Therefore, NTM isolated from sputum specimens often does not warrant treatment and must be interpreted in the context of the clinical, radiologic, and microbiologic criteria.^35^ NTM pulmonary disease must be strongly suspected in a patient with persistent signs and symptoms of TB, positive smear microscopy and a negative Mtb nucleic acid amplification test (NAAT).

In SA, the diagnostic criteria outlined by the ATS/ERS/ESCMID/IDSA NTM guideline^34^ are adopted in clinical practice. These are indicated in Table 2.^2,29,34^

**TABLE 2:** Clinical and microbiologic criteria for diagnosis of non-tuberculous mycobacteria pulmonary disease.

Category	Diagnostic criteria
Clinical	Pulmonary or systemic symptoms.
Radiologic	Nodular or cavitary opacities on chest radiograph, or a high-resolution CT scan that shows bronchiectasis with multiple small nodules. Appropriate exclusion of other diagnoses. Both clinical and radiologic criteria are required.
Microbiologic†	Positive culture results from at least two separate expectorated sputum samples. If the results are nondiagnostic, consider repeat sputum AFB smears and cultures. or Positive culture results from at least one bronchial wash or lavage. or Transbronchial or other lung biopsy with mycobacterial histologic features (granulomatous inflammation or AFB), and positive culture for NTM or biopsy showing mycobacterial histologic features (granulomatous inflammation or AFB), and one or more sputum or bronchial washings that are culture positive for NTM.

*Source*: Daley C, Iaccarino J, Lange C. Corrigendum to: Treatment of nontuberculous mycobacterial pulmonary disease: An official ATS/ERS/ESCMID/IDSA clinical practice guideline. Clin Infect Dis. 2020;71(11):3023. https://doi.org/10.1093/cid/ciaa1062; Griffith DE, Aksamit T, Brown-Elliott BA, et al. An official ATS/IDSA statement: Diagnosis, treatment, and prevention of nontuberculous mycobacterial diseases. Am J Respir Crit Care Med. 2007;175(4):367–416. https://doi.org/10.1164/rccm.200604-571ST; and Haworth CS, Banks J, Capstick T, et al. British Thoracic Society guidelines for the management of non-tuberculous mycobacterial pulmonary disease (NTM-PD). Thorax. 2017;72(Suppl 2):ii1–ii64. https://doi.org/10.1136/thoraxjnl-2017-210927

AFB, acid-fast bacilli; CT, computed tomography; NTM, Non-tuberculous mycobacteria.

† , When two positive cultures are obtained, the isolates should be the same NTM species (or subspecies in the case of *M. abscessus*) in order to meet disease criteria.

Expert consultation should be obtained when NTM are recovered that are either infrequently encountered or that usually represent environmental contamination, such as *M. gordonae*.^36^ Patients who are suspected of having NTM pulmonary disease but do not meet the diagnostic criteria should be followed until the diagnosis is firmly established or excluded. Making the diagnosis of NTM pulmonary disease does not necessitate the use of therapy; this should be a decision based on the potential risks and benefits of therapy for individual patients.^37^

#### Classifying disease severity

Table 3 details how to classify the severity of pulmonary NTM.^2,38^

**TABLE 3:** Classifying pulmonary non-tuberculous mycobacteria disease severity.

Criteria	Non-severe disease	Severe disease
Symptoms	Mild-moderate, no signs of systemic infection (e.g. fever, tachycardia)	Haemoptysis, respiratory failure, signs of systemic infection
Radiological evidence of cavitation/extensive lung disease	None	Present
AFB smear status on respiratory specimen	Smear negative	Smear positive

*Source*: Haworth CS, Banks J, Capstick T, et al. British Thoracic Society guidelines for the management of non-tuberculous mycobacterial pulmonary disease (NTM-PD). Thorax. 2017;72(Suppl 2):ii1–ii64. https://doi.org/10.1136/thoraxjnl-2017-210927; and Sharma SK, Upadhyay V. Epidemiology, diagnosis & treatment of non-tuberculous mycobacterial diseases. Indian J Med Res. 2020;152(3):185–226. https://doi.org/10.4103/ijmr.IJMR_902_20

AFB, acid-fast bacilli.

### Laboratory diagnosis of non-tuberculous mycobacteria

Although there have been significant advancements in laboratory diagnostic methods for the detection of NTM, access to these resources is limited in SA. The microbiology laboratory plays a pivotal role in the diagnosis of NTM infection. Identification of significant NTM species is important to guide the management of disease. It is also important epidemiologically, as species vary according to different geographical locations. In SA, the requirement for further identification and susceptibility testing of NTM must be discussed with the laboratory as this is not offered routinely in all TB laboratories.

#### Specimen collection

It is critical that good quality specimens are obtained to achieve good quality results. NTM species can be isolated from various respiratory and non-respiratory specimens.

Respiratory specimens include:

- Sputum (expectorated or induced)
- Endotracheal aspirates
- Bronchial washings and lavage
- Gastric aspirates.

For disseminated disease, non-respiratory specimens include:

- Blood cultures
- Tissue (including bone marrow biopsy)
- Sterile fluids
- Lymph node biopsies.

#### Specimen processing

Stains for acid-fast bacilli may detect NTM; however, these cannot differentiate NTM from Mtb, nor can NTM subspecies be identified. Therefore, detection by culturing specimens in media is essential for the definite diagnosis of NTM.

Culture of respiratory samples can be performed on both liquid and solid media; however, due to ease of processing in high-throughput laboratories in SA, liquid culture using the Mycobacterial Growth Indicator Tubes (MGIT^TM^) 960 system is preferred.

The time to positivity of culture can be used to further guide whether the NTM is a rapid- or slow-growing mycobacterium. Rapid growers can show growth within 7 days of culture and clinically relevant species include *M. fortuitum, M. chelonae, Mycobacterium smegmatis, M. abscessus* and *Mycobacterium mucogenicum*. Clinically relevant slow growers include MAC, *Mycobacterium haemophilum, M. kansasii,* and *Mycobacterium ulcerans*. These species usually take more than 7 days to show growth.

#### Species identification

Once the specimen has confirmed mycobacterial growth on culture, further serological testing is performed within the routine laboratory to differentiate between Mtb and NTM. Several commercial rapid serological kits that identify the MPT64 antigen (a protein that is secreted by actively growing strains of *M. tuberculosis*) are available. MPT64 antigen is not detected in NTM and Bacillus Calmette-Guerin strains with RD2 deletion; therefore, a positive MPT64 antigen test indicates the presence of Mtb.^39^ It is important to note that rapid antigen testing is part of TB culture laboratory workflow, and therefore does not have to be specifically ordered by clinicians. A potential limitation in MPT64 antigen assays is that false positives have been reported in literature.^1,40^

Once NTM is established in a clinical isolate, clinically relevant isolates should then be identified by molecular methods. There are several commercially available NAATs and mass spectrometry methods for speciation of NTM. In SA, a line probe assay is performed using the GenoType Mycobacterium CM (‘Common Mycobacteria’) and GenoType Mycobacterium AS (‘Additional Species’) kits (Hain Lifescience GmbH, Nehren, Germany). These assays are validated for use from cultured isolates only and cannot be performed directly on specimens.^41^ However, depending on the laboratory setting, these tests may be performed directly on the specimen following consultation with the attending clinical microbiologist.

#### Drug susceptibility testing

In general, NTM antimicrobial susceptibility testing may be performed for clinically significant isolates and in patients who are not responding to treatment. Positive isolates from blood cultures, other sterile body fluids, and skin and soft tissue infections are usually clinically significant.^41^ Requests for susceptibility testing must be discussed with the laboratory as this is not routinely available in all South African TB reference laboratories, and clinically relevant susceptibility breakpoints are only known for certain drugs. For MAC, only azithromycin susceptibility testing is routinely required, and for *M. kansasii*, only rifampicin susceptibility is routinely necessary. Susceptibility testing is recommended in patients that are not responding to standard NTM treatment regimens.

Specialised laboratories may offer phenotypic susceptibility testing on clinically relevant NTMs for which there are established breakpoints.

## Treatment

An essential component in the management of these patients is the early initiation of ART once tolerating medication (preferably after 2 weeks), to ensure success of the treatment.

Where a macrolide is required for the treatment of NTM, azithromycin should be used over clarithromycin, as there are fewer drug-drug interactions, an easier dosing schedule, better tolerance, and equal efficacy.^34^

Treatment for NTM in CWH is the same as for adults with HIV.

### Disseminated non-tuberculous mycobacteria infection

#### Treatment regimens

The cornerstone of therapy for disseminated MAC infection is a macrolide (azithromycin or clarithromycin) together with ethambutol, as a variety of treatment regimens without a macrolide have been proven to be ineffective.^29^ A rifamycin (rifabutin or rifampicin) can be added.^26,42,43^ Patients with a poor response to treatment should be tested for drug resistance.^26^ At least two new agents should be added in resistant disseminated NTM infection. Drugs that should be considered for inclusion are rifamycins, aminoglycosides and quinolones. Macrolide therapy should be continued, using the principle that most patients will have a mixed infection, with some sensitive strains remaining.^26,34^ Clofazimine has been associated with excess mortality in the treatment of disseminated MAC disease and should be used with caution.^29^

Table 4 details treatment regimens for disseminated NTM infection caused by MAC or *M. kansasii*. Other NTM (including *Mycobacterium genavense, Mycobacterium simiae, M. haemophilum, M. chelonae, M. abscessus* and *M. fortuitum*) have been described in HIV and should be treated if found on blood culture in PWH.^29,38^ Guidance on how to treat these infections can be found in the 2007 ATS/IDSA NTM guidelines as well as articles referenced.^26,29,38,44,45,46,47^

**TABLE 4:** Recommended treatment regimens for disseminated non-tuberculous mycobacteria infection in people with HIV.

NTM species	Resistance pattern	Suggested regimen
MAC	Macrolide susceptible	Azithromycin 10 mg/kg per dose (max 500 mg) PO daily **PLUS** Ethambutol 15 mg/kg (max 1.6 g) PO daily **±** Rifampicin 450 mg (< 50 kg) or 600 mg (≥ 50 kg) daily PO **OR** Rifabutin 5 mg/kg (max 300 mg) daily PO†
	Macrolide resistant	**ADD > 2 NEW DRUGS** Amikacin 15 mg/kg (if given daily) or 15–25 mg (if given 3 times a week) IVI **OR** Moxifloxacin 4 mg/kg (max 200 mg if < 45 kg or 400 mg if ≥ 45 kg) daily PO **OR** Levofloxacin 15 mg/kg (max 1000 mg) daily PO **OR** Rifampicin 450 mg (< 50 kg) or 600 mg (≥ 50 kg) daily PO **OR** Rifabutin 5 mg/kg (max 300 mg) daily PO†
*Mycobacterium kansasii*	Rifampicin susceptible	Rifampicin 450 mg (< 50 kg) or 600 mg (≥ 50 kg) daily PO **PLUS** Ethambutol (25 mg/kg daily) PO **PLUS** Azithromycin 10 mg/kg per dose (max 500 mg) PO **OR** Isoniazid 5 mg/kg (max 300 mg) daily PO with pyridoxine 25 mg daily PO
	Rifampicin resistant	Moxifloxacin 4 mg/kg (max 200 mg if < 45 kg or 400 mg if ≥ 45 kg) daily PO **PLUS** Ethambutol 15 mg/kg (max 1.6 g) daily PO **PLUS** Azithromycin 10 mg/kg per dose (max 500 mg) PO **OR** Isoniazid 5 mg/kg (max 300 mg) daily PO with pyridoxine 25 mg daily PO

*Source*: Clinical management of rifampicin-resistant tuberculosis. Updated clinical reference guide. Pretoria: South African National Department of South Africa; 2023; Griffith DE, Aksamit T, Brown-Elliott BA, et al. An official ATS/IDSA statement: Diagnosis, treatment, and prevention of nontuberculous mycobacterial diseases. Am J Respir Crit Care Med. 2007;175(4):367–416. https://doi.org/10.1164/rccm.200604-571ST; Pennington KM, Vu A, Challener D, et al. Approach to the diagnosis and treatment of non-tuberculous mycobacterial disease. J Clin Tuberc Other Mycobact Dis. 2021;24:100244. https://doi.org/10.1016/j.jctube.2021.100244; Sharma SK, Upadhyay V. Epidemiology, diagnosis & treatment of non-tuberculous mycobacterial diseases. Indian J Med Res. 2020;152(3):185–226. https://doi.org/10.4103/ijmr.IJMR_902_20; and Wi YM. Treatment of extrapulmonary nontuberculous mycobacterial diseases. Infect Chemother. 2019;51(3):245–255. https://doi.org/10.3947/ic.2019.51.3.245

IVI, intravenous infusion; max, maximum; *Mycobacterium avium* complex; NTM, non-tuberculous mycobacteria; PO, per os.

† , Ensure rifabutin dose is adjusted for patients on a protease inhibitor.

#### Monitoring and duration of treatment

A clinical response can be expected 2–4 weeks following treatment initiation.^29^ However, this response may be delayed in those with more extensive MAC disease or advanced immunosuppression.^29^ In PWH with a poor clinical response, a repeat blood culture should be obtained 4–8 weeks after initiating treatment.^48^ Treatment can be discontinued when the patient is asymptomatic and has a sustained increase in CD4 count to > 100 cells/µL for ≥ 12 months along with a suppressed HIV viral load.^29^

### Pulmonary non-tuberculous mycobacteria infection

#### Treatment regimens

In the case of MAC pulmonary disease in PWH, a two-drug regimen is recommended over a three-drug regimen, as guidelines recommending a three-drug regimen are mostly from developed countries, where prophylaxis for NTM infection is used, thereby increasing the chance of macrolide resistance. One SA study showed only 2.8% macrolide resistance.^49^ In addition, three-drug regimens have the disadvantage of an increased pill burden and robust evidence for this recommendation is not available.^29,34,38,50^ The recommended treatment regimen is detailed in Table 5.^2,29,34,36,38,51^ There is an option of adding intravenous amikacin 15 mg/kg (if given daily) or 15 mg – 25 mg (if given three times a week) for the first 3 months of treatment when there is severe disease, the patient is failing therapy, or there is concern with oral drug absorption.^2,29,34,38^

**TABLE 5:** Recommended treatment regimens pulmonary infection in people with HIV.

NTM species	Resistance pattern	Suggested regimen
MAC	Macrolide susceptible	Azithromycin 10 mg/kg per dose (max 500 mg) daily PO **PLUS** Ethambutol 15 mg/kg (max 1.6 g) daily PO
	Macrolide resistant	Rifampicin 450 mg (< 50 kg) or 600 mg (≥ 50 kg) daily PO **PLUS** Ethambutol 15 mg/kg (max 1.6 g) daily PO **PLUS** Isoniazid 5 mg/kg (max 300 mg) daily PO **OR** Moxifloxacin 4 mg/kg (max 200 mg if < 45 kg or 400 mg if ≥ 45 kg) daily PO
*Mycobacterium kansasii*	Rifampicin susceptible	Rifampicin 450 mg (< 50 kg) or 600 mg (≥ 50 kg) daily PO **PLUS** Ethambutol 15 mg/kg (max 1.6 g) daily PO **PLUS** Azithromycin 10 mg/kg per dose (max 500 mg) PO **OR** Isoniazid 5 mg/kg (max 300 mg) daily PO
	Rifampicin resistant	Ethambutol 15 mg/kg (max 1.6 g) daily PO **PLUS** Moxifloxacin 4 mg/kg (max 200 mg if < 45 kg or 400 mg if ≥ 45 kg) daily PO **PLUS** Azithromycin 10 mg/kg per dose (max 500 mg) PO **OR** Isoniazid 5 mg/kg (max 300 mg) daily PO

*Source*: Bartlett JG, Pham PA. Johns Hopkins ABX Guide 2012. Jones & Bartlett Publishers; 2011; Daley C, Iaccarino J, Lange C. Corrigendum to: Treatment of nontuberculous mycobacterial pulmonary disease: An official ATS/ERS/ESCMID/IDSA clinical practice guideline. Clin Infect Dis. 2020;71(11):3023. https://doi.org/10.1093/cid/ciaa1062; Griffith DE, Aksamit T, Brown-Elliott BA, et al. An official ATS/IDSA statement: Diagnosis, treatment, and prevention of nontuberculous mycobacterial diseases. Am J Respir Crit Care Med. 2007;175(4):367–416. https://doi.org/10.1164/rccm.200604-571ST; Haworth CS, Banks J, Capstick T, et al. British Thoracic Society guidelines for the management of non-tuberculous mycobacterial pulmonary disease (NTM-PD). Thorax. 2017;72(Suppl 2):ii1–ii64. https://doi.org/10.1136/thoraxjnl-2017-210927; Lange C, Böttger EC, Cambau E, et al. Consensus management recommendations for less common non-tuberculous mycobacterial pulmonary diseases. Lancet Infect Dis. 2022;22(7):e178–e190. https://doi.org/10.1016/S1473-3099(21)00586-7; and Sharma SK, Upadhyay V. Epidemiology, diagnosis & treatment of non-tuberculous mycobacterial diseases. Indian J Med Res. 2020;152(3):185–226. https://doi.org/10.4103/ijmr.IJMR_902_20

max, maximum; MAC, *Mycobacterium avium* complex; NTM, non-tuberculous mycobacteria; PO, per os.

The recommended treatment regimen for other NTM species causing pulmonary disease in PWH is also detailed in Table 5. In PWH with *M. kansasii* pulmonary disease, treatment with rifampicin is recommended.^34^ Pulmonary *M. malmoense* infection is rare in SA and is not linked to HIV infection; however, isolation of this species on a respiratory sample is clinically relevant and should be investigated.^16,29,38^ Treating this species can be difficult and the optimal treatment is unknown; however, some microbiologic improvement has been documented with combinations of isoniazid, rifampicin, and ethambutol, with and without quinolones and macrolides.^29^ *M. scrofulaceum* has been described as a cause of pulmonary NTM disease in HIV, but with new speciation methods, cultures previously identified as *M. scrofulaceum* have been re-identified as *M. gordonae*.^14,29^ Treatment of a true infection with *M. scrofulaceum* would need expert consultation. As *M. gordonae* is likely non-pathological, these should not be acted upon if cultured from a respiratory sample.^2,36^

#### Monitoring and duration of treatment

With pulmonary NTM disease, sputum cultures should be taken 1–2 monthly^34^ and stop once 3 consecutive cultures are negative. Cultures should resume if there is clinical deterioration. At a minimum, a chest X-ray should done before starting treatment and at the end of treatment to document the radiological response to treatment.^2^ If resources allow, a chest computed tomography (CT) scan can be used in place of a chest X-ray, but the increased exposure to radiation should be kept in mind.^2^ NTM treatment should be continued until 1 year post culture conversion together with a clinical response, the start of ART, and HIV viral suppression.^2,29,34^

### Skin and soft tissue non-tuberculous mycobacteria infections

As noted above, the incidence of NTM skin and soft tissue infections is not directly increased in PWH. However, MAC and *M. kansasii* skin and soft tissue infections may represent disseminated disease in patients with advanced HIV infection (CD4 < 100 cells/µL).^52,53^ At the very least, a mycobacterial blood culture should be performed in these patients and they should be treated as for disseminated disease.^29,54,55^ International guidelines or an expert should be consulted when managing PWH presenting with infections due to other NTM species.

### Lymphadenitis

Lymphadenitis is largely seen in immunocompetent people and children.^38,47^ Local NTM lymphadenitis in the context of PWH usually reflects an IRIS reaction and medical therapy is recommended, although no randomised control studies have been done in this specific scenario.^29^ Combination therapy with a macrolide-based regimen should be considered for these patients.^29,38^ Duration of therapy is unknown with durations ranging from 0 to 38 months in individual cases based on clinical response.^32,56,57,58,59^ The addition of steroids should be considered in these patients, especially if the lymphadenitis is extensive or causing complications.^32,56,58^ Ensure disseminated NTM infection is ruled out with a mycobacterial blood culture.

## Adverse drug reactions

Adverse drug reactions are important, especially as patients are on prolonged durations of treatment and frequently have co-morbidities. Table 6 details the most important serious adverse effects in the most commonly used drugs. When in doubt, a trusted source, such as the Liverpool drug interaction online tool (Liverpool HIV Interactions [hiv-druginteractions.org]), should be consulted.^38,47^

**TABLE 6:** Serious adverse effects and monitoring of commonly used drugs in the treatment of non-tuberculous mycobacteria infections.

Drug	Adverse effects	Monitoring
Macrolides	QTc prolongation if given with other QTc-prolonging drugs SJS, TEN Ototoxicity Hepatitis (rarely) Abdominal pain.	Monitor QTc at 2 weeks then monthly if given with other QTc-prolonging drugs Audiogram at baseline and then every 3 months If symptoms/signs of DILI, check liver function tests.
Ethambutol	Optic neuritis ■ Dose-dependent ■ Generally reverses on discontinuation ■ Red-green colour blindness (increased risk with concurrent use of isoniazid).	Eye examination at baseline Monthly visual acuity tests and colour discrimination tests Fundoscopy at baseline and then every 3 months.
Rifampicin/rifabutin	Hepatitis Cytopenia Uveitis.	If symptoms/signs of DILI, check liver function tests Check full blood count.
Isoniazid	Hypersensitivity reaction Hepatitis Peripheral neuropathy.	Monitor for peripheral neuropathy at each visit If symptoms/signs of DILI, check liver function tests.
Amikacin (intravenous)	Nephrotoxicity Ototoxicity ■ Hearing loss, tinnitus, loss of balance ■ Auditory effects more common than vestibular.	Weekly renal function Weekly drug trough level monitoring for the first 4 weeks ■ Should be ‘undetectable’ to ensure no drug accumulation.
Moxifloxacin	Tendinitis, tendon rupture Peripheral neuropathy QTc prolongation CNS effects (dizziness, anxiety, agitation, disorientation, memory impairment, insomnia).	Monitor QTc at 2 weeks then monthly if given with other QTc-prolonging drugs Monitor for peripheral neuropathy, tendinitis, CNS effects at each visit.

*Source*: Pennington KM, Vu A, Challener D, et al. Approach to the diagnosis and treatment of non-tuberculous mycobacterial disease. J Clin Tuberc Other Mycobact Dis. 2021;24:100244. https://doi.org/10.1016/j.jctube.2021.100244; and Sharma SK, Upadhyay V. Epidemiology, diagnosis & treatment of non-tuberculous mycobacterial diseases. Indian J Med Res. 2020;152(3):185–226. https://doi.org/10.4103/ijmr.IJMR_902_20

CNS, central nervous system; DILI, drug induced liver injury; QTc, Corrected QT interval; SJS, Stevens Johnson syndrome; TEN, toxic epidermal necrolysis.

## Immune reconstitution inflammatory syndrome

IRIS is a result of recovery of specific cell-mediated immune response to NTM antigens associated with ART and it most often occurs within 3 months of ART initiation.^60^ IRIS can be either paradoxical (a patient already on NTM treatment and symptoms worsen when starting ART) or unmasking (NTM symptoms only develop when starting ART). IRIS is an important concern for severely immunosuppressed patients (CD4 < 100 cells/µL) initiating ART.^32,61,62^ Other risk factors for IRIS include a low body mass index, anaemia, and a raised ALP.^62,63^

Pulmonary symptoms and lymphadenopathy represent the most common manifestations of MAC-IRIS,^29^ and it should therefore be considered in the differential diagnosis of a patient who presents with fever and/or worsening or new symptoms soon after initiating ART.^62^ Paradoxical IRIS reactions to disseminated disease often present with abdominal involvement, perhaps reflecting the large organism burden in the gut and abdominal lymph nodes.^32^ Systemic symptoms are again prominent.

The disease course varies widely in MAC-IRIS. Patients can have a benign self-limiting course, some patients clear the infection without MAC treatment and with immune restoration alone, whereas others have several relapses or a severe protracted disease lasting years, despite ART, MAC treatment, and corticosteroids.^32,64,65^

Patients who develop mild-to-moderate symptoms typical of IRIS should receive initial treatment with a short-course of non-steroidal anti-inflammatories.^66^ If IRIS symptoms do not improve or symptoms are severe, short-term (4–8 weeks) systemic corticosteroid therapy has been anecdotally successful in reducing symptoms and morbidity.^48,66,67^ This should be initiated after resistance to treatment and other co-infections, such as TB, have been excluded. If prolonged corticosteroids are required, we recommend discussing the case with an Infectious Diseases specialist.

Prednisone 1 mg/kg per day (or equivalent) for patients with MAC-IRIS and significant symptoms (e.g. abdominal pain), can be initiated and then weaned according to symptom response. If the patient is on rifampicin, then prednisone should be increased to 1.5 mg/kg per day initially due to the drug interactions.^67^ ART should not be interrupted.

## Prevention

NTM are ubiquitous organisms found in the environment, including in municipal tap water supply^68^ and soil.^69^ It is not possible to avoid all environmental exposures^70,71,72^; therefore, the most effective NTM prevention strategy in PWH is early diagnosis of HIV, initiation of ART and retention in care with viral suppression and immunological reconstitution.^29,38^

NTM have a lipid-rich impermeable outer membrane, making them resistant to commonly used disinfectants.^29,69^ They can persist in municipal water systems and are not killed by chlorine concentrations used for disinfection of household water.^69^ Their ability to form biofilms allows them to adhere to pipe walls and withstand being flushed out by water flow. In addition to water sources, NTM such as MAC are also found in high concentrations in peat-rich potting soils.^70^

While interventions to avoid household and environmental exposure to NTM in high-risk populations have been advocated,^38^ there are currently no evidence-based strategies to avoid exposure in these settings.^29,71^

### Infection control

Infection occurs via inhalation of aerosolised droplets containing NTM from environmental sources, such as showers and hot tubs, into the lungs.^38,73^ Transmission-based infection and prevention control (IPC) precautions are currently not warranted in the management of patients with NTM disease, as human-to-human transmission is thought to be uncommon.^20,29,38,74,75^

Outbreaks and pseudo-outbreaks related to environmental exposure of NTM have occurred in hospital settings, and environmental IPC principles must be applied routinely in all healthcare settings.^29,38^ Unsterile municipal tap water remains an important source of NTM infection.^29^ Tap water must not be used to wash open wounds or in the operating room, and indwelling vascular catheters, dialysate used for haemodialysis, and endoscopes must not be contaminated with tap water.^29,38^

### Prophylaxis

Routine primary prophylaxis for NTM is not recommended in PWH, with the caveat that ART is promptly commenced.^29,38,73^ Primary prophylaxis, however, could be considered in consultation with an Infectious Diseases specialist in patients who are on salvage HIV treatment options. The chemoprophylaxis of choice is azithromycin,^29,38,71,72,73,76^ which is favoured because of the ease of weekly dosing (1250 mg per week), cost effectiveness, and fewer drug-drug interactions.^29,71^ Alternative options include clarithromycin or rifabutin. Active TB must be excluded prior to commencing rifabutin.^38^ Combination chemoprophylaxis strategies are not recommended because of the risk of increased side effects and potential for drug-drug interactions.^29^ Prophylaxis may be discontinued in the event of sustained suppression of HIV viral load for > 3 months.

The current consensus is that there is also no need for secondary prophylaxis after completion of treatment.

## Conclusion

The management of NTM in PWH requires an integrated approach, including early detection, appropriate antimicrobial therapy, and associated monitoring of clinical response. ART plays a pivotal role in improving immune function, to reduce the incidence, severity and complications. Choice of antibiotics is guided by species identification and susceptibility testing and is important to achieve eradication of the pathogen. Regular monitoring for adverse effects and drug interactions is an important component of the treatment plan. Adherence to ART and antibiotic treatment is essential to improve long-term prognosis.
